# Peritoneal invasion of prostate cancer directly confirmed during robot‐assisted radical prostatectomy

**DOI:** 10.1002/iju5.12509

**Published:** 2022-07-19

**Authors:** Takehiro Ohyama, Masaki Shimbo, Kenji Komatsu, Fumiyasu Endo, Naoki Kanomata, Kazunori Hattori

**Affiliations:** ^1^ Department of Urology St. Luke's International Hospital Tokyo Japan; ^2^ Department of Pathology St. Luke's International Hospital Tokyo Japan

**Keywords:** perineal invasion, prostate cancer, robot‐assisted radical prostatectomy

## Abstract

**Introduction:**

Several studies have been published on direct rectal invasion in patients with advanced metastatic prostate cancer, but few have directly confirmed intraoperative invasion of prostate cancer into the peritoneum.

**Case presentation:**

We report the case of a 73‐year‐old man with prostate cancer who exhibited peritoneal invasion during robot‐assisted radical prostatectomy. His prostate‐specific antigen level fell to 0.38 ng/mL after surgery; he was therefore prescribed radiation and androgen‐deprivation therapies that controlled the cancer for more than 1 year.

**Conclusions:**

We encountered a case showing direct peritoneal invasion of prostate cancer during robot‐assisted radical prostatectomy. If invasion of the seminal vesicle is suspected, the vesicorectal fossa should be examined during robot‐assisted radical prostatectomy. Preoperative confirmation by diffusion magnetic resonance imaging of the peritoneum is also useful.

**Abbreviations & Acronyms**
CAB: combined androgen blockade
CT: computed tomography
ePLND: extended pelvic lymph node dissection
MRI: magnetic resonance imaging
PSA: prostate‐specific antigen
RARP: robot‐assisted radical prostatectomy

**Keynote message**

During RARP, we experienced a case of prostate cancer with direct peritoneal invasion. If invasion of a seminal vesicle is suspected, preoperative diffusion MRI may be useful to confirm invasion into the peritoneum and vesicorectal fossa during RARP.

## Introduction

Although direct invasion of the rectum^1^ has been described in patients with advanced metastatic prostate cancer, no report on intraoperatively confirmed peritoneal invasion has appeared. We report a case of peritoneal invasion confirmed by RARP.

## Case presentation

**Fig. 1:**
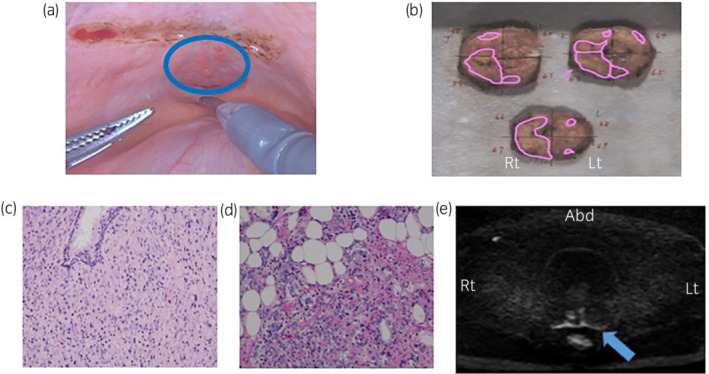
(a) Intraoperative endoscopic findings. The multiple central nodules indicate peritoneal invasion. The blue circle denotes the lesion. (b) Prostate specimen; the pink area represents the area invaded by cancer. The image shows cancer in the right lobe. (c) Prostate specimen stained with hematoxylin and eosin (10×). (d) Peritoneal specimen stained with hematoxylin and eosin showing prostate cancer invasion (10×). (e) Diffusion‐weighted image. The blue arrow denotes a high peritoneal signal.

A 73‐year‐old man visited us for evaluation and treatment of decreased urine flow. His PSA level was 18.72 ng/mL and a digital rectal examination revealed an enlarged prostate that was stony hard in the right peripheral zone, but barely mobile. Pelvic MRI revealed suspicious lesions, mainly in the right lobe of the prostate gland, along with seminal vesicle invasion. PI‐RADs v2^2^ was category 5. A transperineal prostate biopsy led to a histological diagnosis of prostate cancer with a Gleason score of 4 + 5 = 9. Neither CT nor bone scintigraphy revealed metastasis; we thus diagnosed cT3bN0M0 disease. After discussion with the patient, we performed RARP with ePLND. The main radical prostatectomy steps were similar to those of Menon,^3^ with some modifications. We checked the vesicorectal fossa, and observed many unusual nodules on the peritoneum (Fig. 1a). Atypically, the peritoneum was very hard and could not be easily separated. As adhesion caused by tumor invasion was marked, we changed to an anterior approach. During transection of the bladder neck, the right posterior wall was very hard; we thus suspected cancer invasion. The right lobe of the prostate specimen was mostly occupied by cancer (Fig. 1b). Pathologically, the tumor was an adenocarcinoma (Fig. 1c) with a Gleason Score of 5 + 4, and a positive resection margin on the base associated with lymph node metastasis (3/26 nodes). An adenocarcinoma involving the peritoneum (Fig. 1d) was pathologically confirmed. Although no complication developed after RARP, the postoperative PSA level was 0.39 ng/mL at 1 month and 0.53 ng/mL at 2 months. We prescribed adjuvant radiotherapy (66 Gy to the prostate fossa) and continuous androgen deprivation therapy (LHRH monotherapy). There was no biochemical (PSA not detected) or radiographic evidence of recurrence at 18 months after surgery.

## Discussion

We observed a rare case of prostate cancer directly invading the peritoneum. While surgical treatment for high‐risk prostate cancer is becoming more common,^4^ this was an extremely rare finding. Peritoneal invasion is seldom found intraoperatively in prostate cancer, although it is sometimes encountered in bladder cancer.^5^ We summarize two reports of peritoneal invasion, including our case in Table 1.^6^ In both cases, the PSA level was less than 20; however, the Gleason score was high. Labanaris *et al*. confirmed peritoneal invasion by laparoscopy, and biopsy confirmed the diagnosis. The patient was treated with CAB. Therefore, our case is the first report of RARP treatment for prostate cancer with peritoneal invasion. As bone and lymph nodes are the major metastatic sites of prostate cancer, there are also cases in which organ metastasis (to the lungs, liver, brain, and gastrointestinal tract) is detected at the time of the initial diagnosis. In a report of 1589 autopsied cases of prostate cancer, peritoneal and mesenteric metastases were found in 7.0% and 1.1% of cases, respectively.^7^ There have been reports of prostate cancer peritoneal metastasis, including cases of recurrence at the port site.^8^ In our case, the cancer is thought to have invaded the peritoneum directly, implying that high‐grade prostate cancer can directly invade the peritoneum and eventually lead to peritoneal dissemination. In cases of intra‐abdominal metastasis of prostate cancer, direct invasion, such as in this case, may lead to peritoneal dissemination. Although peritoneal dissemination of prostate cancer is not common, it can reach the peritoneal space through direct invasion from the vesicorectal fossa as in this case, in addition to bloody metastasis due to cancer progression.

**Table 1:** Summary of cases in which a peritoneal invasion was identified intraoperatively

Author, year	Gleason score	Other metastases	PSA at diagnosis of peritoneal invasion (ng/mL)	Methods to confirm peritoneal invasion	Treatment of the primary site
1. Labanaris,^6^ 2013	5+4	No	13.3	Laparoscopy	CAB
2. Our case, 2022	5+4	No	18.72	RARP	RARP

In addition, preoperative imaging showed mainly seminal vesicle invasion; it was difficult to identify peritoneal invasion, but a retrospective review revealed a linear area of high‐intensity along the peritoneum on the diffusion‐weighted MRI image, which implied direct invasion of the peritoneum (Fig. 1e). Although such cases are rare, and few imaging studies are available, MRI may be helpful. We expect to encounter more high‐risk cases in the future, and consider that preoperative MRI confirmation is very important in such cases.

The indications for surgery in cases of peritoneal invasion of prostate cancer are controversial. In this case, if the peritoneal invasion had been recognized in advance, we might not have performed RARP. However, in the absence of metastatic findings and based on the diffusion MRI results and dysuria, RARP was performed. The cancer is currently being controlled and urinary function is good. We anticipate applying multimodal treatment during follow‐up (adjuvant radiation and adjuvant hormonal therapy). We will carefully follow the patient's progress.

## Conclusion

We report a case of prostate cancer peritoneal invasion discovered during RARP. If periprostatic invasion is suspected preoperatively, especially in patients with suspected seminal vesicle invasion, observation of the vesicorectal fossa during RARP is essential. Although peritoneal invasion is rare, MRI diffusion imaging may reveal seminal vesicle invasion, as in our case.

## Author contributions

Takehiro Ohyama: Conceptualization; visualization; writing – original draft. Masaki Shimbo: Supervision; writing – review and editing. Kenji Komatsu: Supervision. Fumiyasu Endo: Supervision. Naoki Kanomata: Supervision. Kazunori Hattori: Supervision.

## Conflict of interest

The authors declare no conflict of interest.

## Approval of the research protocol by an Institutional Reviewer Board

Not applicable.

## Informed consent

Not applicable.

## Registry and the Registration No. of the study/trial

Not applicable.
